# High intraspecific ability to adjust both carbon uptake and allocation under light and nutrient reduction in *Halimium halimifolium* L.

**DOI:** 10.3389/fpls.2015.00609

**Published:** 2015-08-07

**Authors:** Frederik Wegener, Wolfram Beyschlag, Christiane Werner

**Affiliations:** ^1^Ecosystem Physiology, University of FreiburgFreiburg, Germany; ^2^AgroEcosystem Research, Bayreuth Center of Ecology and Environmental Research (BayCEER), University of BayreuthBayreuth, Germany; ^3^Experimental and Systems Ecology, University of BielefeldBielefeld, Germany

**Keywords:** ^13^CO_2_, pulse labeling, stable isotopes, respiration, carbon allocation, growth, shade, nutrients

## Abstract

The allocation of recently assimilated carbon (C) by plants depends on developmental stage and on environmental factors, but the underlying mechanisms are still a matter of debate. In the present study, we investigated the regulation of C uptake and allocation and their adjustments during plant growth. We induced different allocation strategies in the Mediterranean shrub *Halimium halimifolium* L. by a reduction of light (*Low L* treatment) and nutrient availability (*Low N* treatment) and analyzed allocation parameters as well as morphological and physiological traits for 15 months. Further, we conducted a ^13^CO_2_ pulse-labeling and followed the way of recently assimilated carbon to eight different tissue classes and respiration for 13 days. The plant responses were remarkably distinct in our study, with mainly morphological/physiological adaptions in case of light reduction and adjustment of C allocation in case of nutrient reduction. The transport of recently assimilated C to the root system was enhanced in amount (c. 200%) and velocity under nutrient limited conditions compared to control plants. Despite the 57% light reduction the total biomass production was not affected in the *Low L* treatment. The plants probably compensated light reduction by an improvement of their ability to fix C. Thus, our results support the concept that photosynthesis is, at least in a medium term perspective, influenced by the C demand of the plant and not exclusively by environmental factors. Finally, our results indicate that growing heterotrophic tissues strongly reduce the C reflux from storage and structural C pools and therefore enhance the fraction of recent assimilates allocated to respiration. We propose that this interruption of the C reflux from storage and structural C pools could be a regulation mechanism for C translocation in plants.

## Introduction

The first step in understanding plant carbon (C) allocation is to investigate the translocation of recent photosynthates. How plants partition recently fixed C among organs and biochemical fractions is likely to be as important to whole plant performance and ecology as photosynthesis itself (Poorter and Sack, 2012). Photosynthetic C isotope discrimination can be used to trace the fate of C from plant to ecosystem scale (see Dawson et al., 2002) but the natural C isotope signals are relatively small. Pulse-labeling with stable or radioactive C isotopes provides a more efficient tool for a detailed analysis of C allocation in plants (for a recent review see Epron et al., 2012). It allows to investigate very fast translocation of C after photosynthetic fixation (Atkin, 2015). In a field study on grasses and shrubs, Carbone and Trumbore (2007) found that 48–61% of the recovered label was respired within 24 h across seasons and plant types.

Carbon allocation strongly depends on environmental factors. In ^13^CO_2_ pulse-labeling experiments, drought was shown to distinctly reduce belowground C transfer and to weaken the link between plant and bacterial C turnover (Ruehr et al., 2009; Barthel et al., 2011; Fuchslueger et al., 2014). In contrast, reduced light did not affect the rate of belowground C transfer in pine (Warren et al., 2012). Elevated CO_2_ concentration (Rouhier et al., 1996; Mikan et al., 2000) as well as a sudden temperature drop (Barthel et al., 2014) were shown to enhance root production, root respiration and C transfer to microbial biomass. For herbaceous plants Inauen et al. (2012) showed a higher accumulation of starch and sugars in leaves under enhanced CO_2_ concentrations, reflecting an imbalance between carbon sources and sinks. However, better knowledge is needed regarding the adjustment of C allocation in response to environmental conditions independent from phenology (Epron et al., 2012).

Compared to light or CO_2_, which directly effects photosynthesis, nutrient limitation effects both C sinks and C sources within the plant. In a meta-analysis, Poorter et al. (2012) found in a comparison with various environmental factors (e.g., water availability, CO_2_ concentration, temperature, irradiance, nutrients) that the fraction of leaves from whole-plant mass (LMF) increases most strongly with nutrients and decreases most strongly with light. In contrast to long-term C allocation, to our knowledge there are no studies investigating the fate of recently fixed C in the plant response to nutrient availability via ^13^C pulse labeling. Fatichi et al. (2014) stated that until now, no study was able to clearly quantify the impact of nutrient limitation on sink (growth) vs. source activity (C assimilation). Moreover, most studies define plants as two- (above- and belowground) or three-part systems (leaf, stem, root) which does not allow an investigation of C allocation into different leaf and root classes, which may vary in their source and sink strength.

Despite the increase in knowledge on C allocation in the last decades, there is still a lack of mechanistic understanding of plant growth. Direct control of C sinks via environmental factors was recently discussed to be more important than “source control” via photosynthetic C gain (Körner, 2013; Fatichi et al., 2014). Water- or temperature-limited plants often show a reduction of growth before photosynthesis declines, resulting in an increase of C storage (e.g., Muller et al., 2011; Sala et al., 2012). Therefore, Körner (2013) suggested that environmental factors first act on sink activity than on source activity. Nevertheless, all existing global vegetation models describe plant growth as directly driven by photosynthesis (Fatichi et al., 2014), showing the need of further research on the hierarchy of plant growth.

The aim of this study was to investigate the phenotypic plasticity of C allocation and its underlying components under varying growth conditions. Therefore, we induced changes in allocation by light and nutrient reduction in the Mediterranean shrub *Halimium halimifolium*. We measured morphological and physiological traits as well as biomass allocation for 15 months. Further, we conducted a ^13^CO_2_ pulse-labeling and followed the fate of recently assimilated C to eight tissue classes and respiration for 13 days. Through the combination of short and long-term allocation monitoring we intend to gain new insights into the regulation of C allocation and plant growth.

## Materials and methods

### Experimental design

Our study species was *Halimium halimifolium* L., an evergreen, Mediterranean subshrub from the *Cistaceae* family. All plants were grown in a well-ventilated walk-in plant growth chamber. Artificial light was provided from 09:00 to 21:00 h for all treatments. Air temperature was 23 and 15°C during the light and dark periods, respectively. Relative air humidity was 60% and photosynthetic photon flux density (PPFD) at substrate surface height was 370 μmol m^−2^ s^−1^. Seven months after sowing plants reached a mean dry weight of 0.76 g (±0.04; SE). At this point 78 plants were transferred into 3 L pots filled with sieved sand and the light and nutrient treatments (described below) were implemented in the growth chamber. PPFD was reduced to 160 μmol m^−2^ s^−1^ in the shade treatment (*Low L*). Shade was provided by neutral density shade cloth. To prevent differences in illumination plants were rotated weekly during the whole experiment. *Control* and *Low L* plants were fertilized weekly with 75 mL of one-fourth strength [3.7 mM nitrogen (N)] of modified Hoagland's fertilizer solution (Peperkorn et al., 2005). The reduced nutrient treatment (*Low N*) was not fertilized in the first 3 months of the experiment and thereafter once a week with 75 mL 1/16 strength (0.9 mM N) of modified Hoagland's solution. Plants were watered with demineralized water to maintain optimal water availability. The volume of water was equal per plant within each treatment but adjusted over time with plant growth. Subsets of plants were destructively harvested at the beginning (0) and after 4, 6, and 12 months of treatments to determine dry weights of plant tissues. Further, the leaf area of fresh leaves was measured (Image Analysis System, DeltaT Devices Ltd., Cambridge, UK) in subsamples of each plant. Together with the corresponding dry weights these data were used to calculate the specific leaf area (SLA, leaf area per leaf dry weight) and the leaf area ratio (LAR, total leaf area per plant dry weight). None of the plants reached the reproductive phase during the experiment.

### Pulse labeling

The ^13^CO_2_ pulse-labeling was conducted 15 months after inducing the treatments with 24 plants (eight plants per treatments and control). Before labeling, the pots were wrapped in two layers of plastic bags and sealed with rubber bands and Terostat (Henkel, Düsseldorf, Germany) around the stem to prevent diffusion of the ^13^C labeled CO_2_ into the soil. The pots were encased by an airtight transparent plastic tent with a volume of approximately 1 m^3^ inside the growth cabinet. Labeling started at 09:45 with 99% ^13^CO_2_ (Linde, Höllriegelskreuth, Germany) supplied as a short pulse (4 h). To stabilize the temperature, humidity, and CO_2_ conditions during the labeling, the air was pumped (10 L/min) through a buffer (200 L) and back to the tent. A cavity ring-down spectrometer (CRDS, G2101-I, Picarro Inc., USA) was connected to the circulating air stream to trace the ^12^CO_2_/^13^CO_2_ concentration in the tent. The use of a powerful fan ensured good mixing of the air inside the tent. We used a strong labeling pulse to ensure high signals in plant bulk material. During 8 min after injecting the label the ^13^C proportion in the tent rose to the maximum value of 88 atom%. During the initial 30 min of labeling the averaged CO_2_ concentration was at 2800 ppm. Then the values decreased to 60 atom% ^13^C and 550 ppm CO_2_ during the 4 h labeling period. Labeling was stopped after 4 h at 13:45, the tent was removed and the growth room was ventilated with outside air. Immediately after labeling we took leaf subsamples from each plant to determine the amount of initially fixed ^13^C. Two plants of each treatment were harvested 5, 18, 30 h, and 13 days (310 h) after labeling (*n* = 2). Plant material was divided into eight tissue classes: First generation leaves (large leaves attached to the main stem, already present at the beginning of the experiment), second generation leaves (emerged after first generation leaves, smaller, mainly attached to lateral shoots), young leaves (expanded but not fully mature), emerging leaves (small leaves, just sprouting), lateral shoots, stem, main root (woody root fraction), and fine roots (non-woody roots). Sand was washed off the roots before drying. Subsamples of each tissue class were frozen in liquid nitrogen and freeze-dried. The freeze-dried material was weighed, ground to powder in a ball mill (MM 2000, Retsch GmbH, Haan, Germany) and stored over silica-gel.

### Leaf level gas-exchange and chlorophyll fluorescence

Measurements were conducted using a portable gas-exchange analyzer (GSF-3000; Walz, Effeltrich, Germany) with the measuring head 3055−FL (enabling chlorophyll fluorescence measurements) for single leaf measurements. Gas-exchange parameters and effective quantum yield were measured simultaneously in the plant growth chamber at growth conditions (25°C, 60% RH and 370 or 160 μmol m^−2^ s^−1^ PPFD) and CO_2_ concentration was set to 400 ppm controlled by the integrated gas mixing unit of the GFS 3000. Measurements were conducted close to the harvesting dates (± 2 days) and evenly distributed over the light period. Gas-exchange parameters were measured on fully expanded, mature leaves. Light-response curves of photosynthesis were measured at the sampling point after 6 months. All gas exchange parameters are reported relative to leaf surface area.

### Whole plant gas-exchange chamber

After pulse labeling one plant of each treatment and control was randomly selected and placed in a whole-plant gas-exchange chamber for 13 days. Three custom build soil-canopy chambers were interfaced with a CRDS (G2101-I, Picarro Inc., USA) to measure ^12^CO_2_/^13^CO_2_ and water vapor concentrations. The chambers were made of 300 mm-diameter acryl glass tubes (Kahmann und Ellerbrock, Bielefeld, Germany). The soil compartment, a bottom closed cylinder (height = 250 mm), was designed to fully enclose the planting pot. The canopy chamber was a top closed cylinder (height = 500 mm). The compartments were separated by a two-piece plate with a gasket between the two sides, which surrounded the stem base. To minimize boundary layer effects the canopy chambers were equipped with two fans and the soil chambers with one fan. A quantum sensor was positioned at crown height within the chamber and small data loggers (Hobo U12, Onset, Bourne, MA, USA) with integrated sensors were placed in the chambers to monitor the temperature in both compartments. The chambers were placed in a climate cabinet set up with growth conditions of the plants. Soil and canopy chambers were permanently flushed at a rate of 1 and 1.5 L/min, respectively, in order to maintain steady state conditions. A solenoid valve system switched between three plant chambers (canopy or soil compartments) and reference (inlet) air to be analyzed by the CRDS. Each gas stream was measured for 15 min but only the last 5 min were averaged and used as a data point. The isotopic composition of the respired CO_2_ was calculated using mass balance equations. During the first days after labeling we predominantly analyzed rhizosphere respiration to determine the peak dynamics in a high time resolution. Because of the missing first days we decided to exclude the measured aboveground flux data from the present study.

### Isotope ratio mass spectrometry and calculations

δ^13^C of dark-respired CO_2_ from tissues was analyzed by the in-tube incubation method as described in Werner et al. (2007). In brief, leaf, stem, main root, and fine root segments were transferred into glass vials and immediately flushed with CO_2_-free air. Samples were left to respire in the dark for precisely 3 min. After that the isotopic composition of CO_2_ was measured using an isotope ratio mass spectrometer (IRMS, Isoprime; Elementar, Germany) interfaced to an autosampler (Microgas, GV, Manchester, UK). The freeze-dried solid samples were combusted in an elemental analyzer (EuroEA, HEKAtech GmbH, Wegberg, Germany) and analyzed in the same IRMS (for a detailed description of the system and measuring procedure, see Werner et al., 2009).

Isotopic composition is presented in δ-notation in per mill units (‰) with respect to the Vienna Pee Dee Belemnite (VPDB) standard or in the percentage contribution of ^13^C to the total number C atoms (atom%):

(1) $${}^{13}\text{C }(\text{atom}\%)\;=\;\frac{{}^{13}C}{{}^{12}C\;+{\;}^{13}C}\;\times \;100$$

We calculated ^13^C_excess_, the increase in ^13^C atoms due to pulse-labeling, using the following equation:

(2) $${}^{13}{\text{C}}_{\text{excess}}\;(\text{atom}\%)\;={\;}^{13}{\text{C}}_{\text{sample}}\;(\text{atom}\%){-}^{13}{\text{C}}_{\text{natural}}\;(\text{atom}\%)$$

with ^13^C_sample_ being the atom% of the labeled sample and ^13^C_natural_ being the atom% of samples taken before labeling. The labeling derived ^13^C amount (in mg ^13^C) per plant or organ was calculated as follows:

(3) $${}^{\text{13}}{\text{C}}_{\text{amount}}\;\left(\text{mg}\right)\;=\;{\;}^{13}{\text{C}}_{\text{excess}}\;\times \text{ C pool }(\text{mg})$$

where, C pool is the total C pool size of the organ or whole plant. The proportion of carbon allocated to a tissue or respiration (^13^C_label_) was calculated based on the amount of initially fixed ^13^C at the end of labeling:

(4) $${}^{\text{13}}{\text{C}}_{\text{label}}\;(\%)\;=\;\frac{{}^{13}\text{C amount }(\text{sample})}{{}^{\text{13}}\text{C amount }(\text{initially fixed})}\;\times \text{100}$$

The initially fixed ^13^C_amount_ was calculated by ^13^C values from leaf subsamples taken immediately after labeling and leaf biomass data from the harvests. In our experimental setup this provided the best available estimate of ^13^C assimilation during the labeling pulse, although a small fraction of label derived ^13^C was already respired and transported out of the leaves when the tent was removed. However, by summing up ^13^C_label_ from all tissues at the first sampling point after 5 h (when respiratory loss was presumably low) we reached values near 100% which shows a good accuracy. Further, Lattanzi et al. (2012) estimated that the underestimation of initial fixed C is less than 10% for labeling periods < 6 h.

To estimate mean residence and half-life times of the respiratory substrate pool for leaf and rhizosphere respiration, the following exponential decay function was used:

(5) $$C(t)\;=\;C_{0}\;e^{\left(-kt\right)}$$

where, *t* is the time after the ^13^C peak, *C*_0_ represents δ^13^CO_2_ at peak time, *k* is the rate constant of tracer loss and *C*(*t*) the δ^13^CO_2_ at time t. The mean residence time (MRT) of ^13^C was then calculated as MRT = 1/*k*, and the half-life time (*t*) was calculated as *t* = ln(2)/*k*.

### Statistics

One-Way analysis of variance (ANOVA) followed by Fisher's least significant difference (LSD) test with Bonferroni correction was used to compare treatments (shown in Table 1). We used Levene's test to test for homogeneity of variance. All variances were homogenous. Normal distribution was tested by Shapiro–Wilk test. All variables were normally distributed. Significant differences are indicated at *p* < 0.05. Statistical analyses were conducted in R 2.15.3 (R Development Core Team, 2013). Regression analyses were carried out in Sigma Plot 12 (Systat Software Inc., Chicago, IL, USA).

**Table 1:** **Effect of light and nutrient reduction on *H. halimifolium***.

	**Treatment**		
	**Control**	**Low N**	**Low L**
PPFD (μmol m^−2^ s^−1^)	370	370	160
N supply (mM N)	3.7	0.9	3.7
Height (cm)	33.1 ± 1.6 a	21.2 ± 1.8 b	47.2 ± 2.5 c
DW (g)	30.2 ± 1.9 a	11.8 ± 0.8 b	30.2 ± 2.3 a
Leaf area (cm^2^)	779 ± 73 a	186 ± 13 b	1012 ± 87 c
Root/shoot	0.81 ± 0.05 a	2.00 ± 0.12 b	0.74 ± 0.05 a
SLA (m^2^ kg^−1^)	5.79 ± 0.01 a	5.6 ± 0.02 b	7.98 ± 0.01 c
LAR (m^2^ kg^−1^)	2.55 ± 0.1 a	1.58 ± 0.06 b	3.34 ± 0.08 c
LMF	0.44 ± 0.02 a	0.28 ± 0.01 b	0.42 ± 0.01 a
RMF	0.45 ± 0.02 a	0.66 ± 0.01 b	0.42 ± 0.01 a
SMF	0.11 ± 0.01 a	0.05 ± 0.01 b	0.16 ± 0.01 c
C/N	54.5 ± 2.1 a	74.8 ± 5.4 b	49.7 ± 2.6 a
ΔF/Fm′	0.46 ± 0.01 a	0.36 ± 0.03 b	0.58 ± 0.05 c

Changed growing conditions are shown above the broken line: PPFD measured at substrate surface and nitrogen (N) concentration in fertilizer solution. Masses, areas, and C/N-values were measured at the end of the experiment (after 15 months). Chlorophyll fluorescence was measured during the experiment (after 12 months). ΔF/Fm′ was measured at growing light conditions (370 or 160 μmol m^−2^ s^−1^). Different letters indicate significant differences (P < 0.05). Data are means (n = 8 ± SE; Chlorophyll fluorescence n = 4 ± SE).

Due to space limitation, we used only two replicates per harvest and treatment in the pulse labeling experiment and therefore did not run further statistical analyses.

## Results

### Changes in morphological and physiological traits and biomass allocation

**Figure 1:**
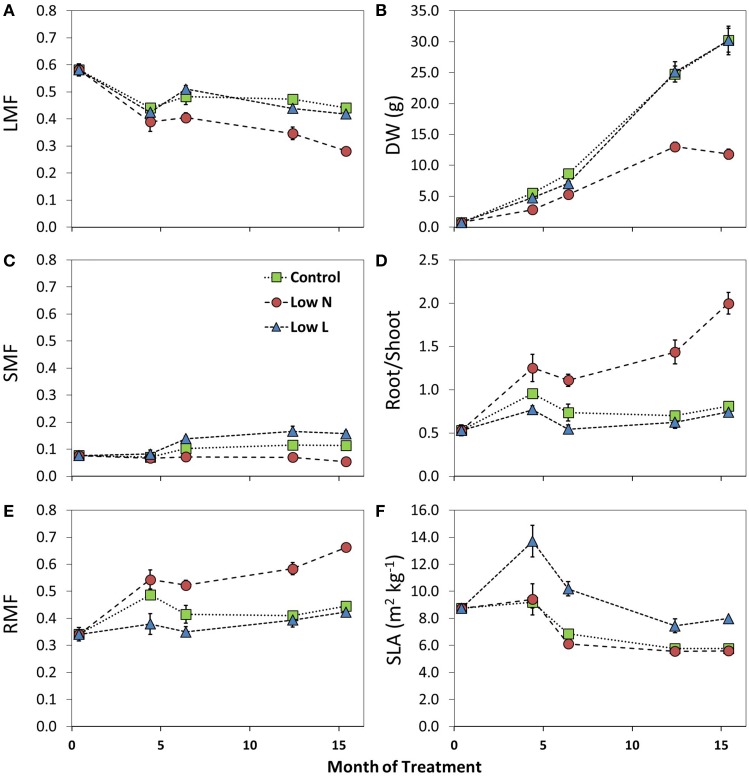
**Changes in leaf mass fraction (LMF, A), stem mass fraction (SMF, C), root mass fraction (RMF, E), dry weight (DW, B), Root/Shoot ratio (D), and specific leaf area (SLA, F) during the 15 months of treatment (*n* = 3–8 ± SE)**. For statistical comparisons between treatments after 15 months see Table 1.

**Figure 2:**
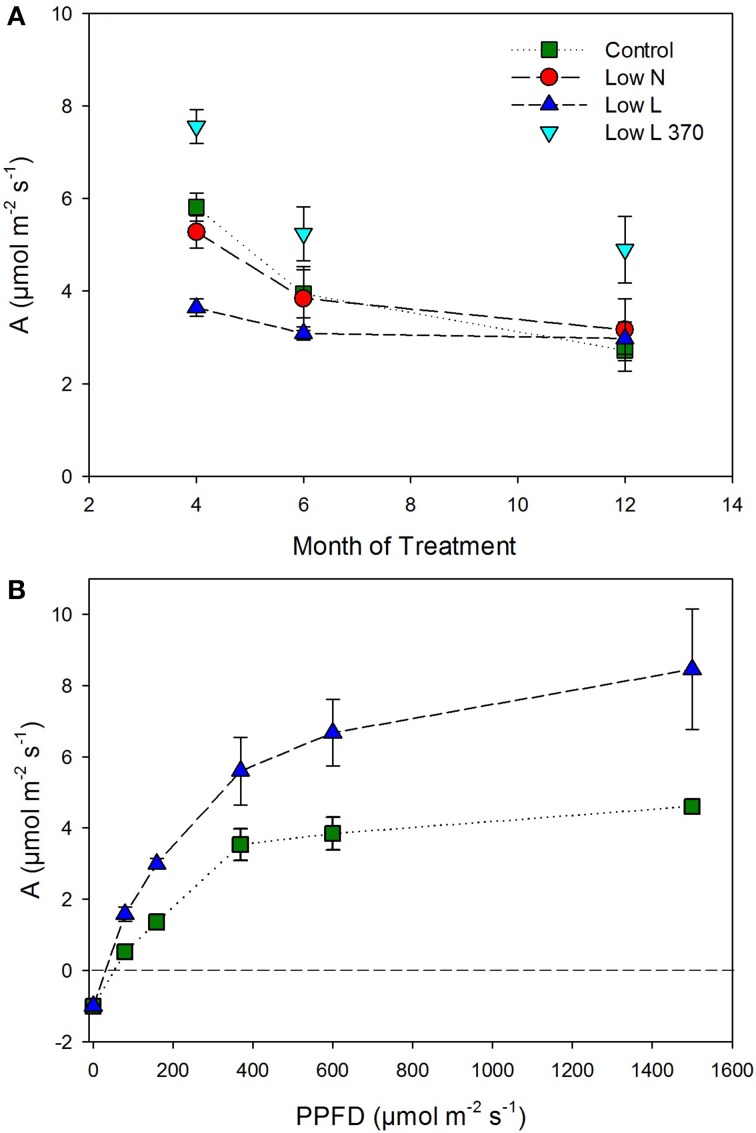
**(A)** Photosynthetic rate (A) under experimental conditions (control and *Low N* 370 μmol m^−2^ s^−2^; *Low L* 160 μmol m^−2^ s^−2^) and additionally for *Low L* plants at 370 μmol m^−2^ s^−2^ (*n* = 4 ± SE). **(B)** Light-response curves of photosynthesis for *Low L* and control plants, measured after 6 months of treatment (*n* = 3 ± SE).

We found a high intraspecific variability of biomass allocation induced by 15 months of nutrient or light reduction (Figure 1). *Low L* and *control* plants showed slightly decreasing leaf mass fractions (LMF) and slightly increasing root mass fractions (RMF). In contrast, the RMF of *Low N* plants nearly doubled at the expense of LMF (Figure 1). Further, the *Low N* treatment significantly reduced overall biomass and plant height (Table 1, Figure 1). *Low L* plants showed a characteristic response to shade with a 40% increased specific leaf area (SLA) and shoot elongation compared to control plants. Although the light was reduced by 57% the dry weight and photosynthetic rate (A) were equal to control plants at the end of the experiment (Figures 1B, 2A). *Low L* plants showed a higher photosynthetic rate compared to control plants when measured under the same light conditions as control plants (Figure 2A, Table S1 in Supplementary Material). Further, light-saturated photosynthesis and effective quantum yield (ΔF/Fm′) were clearly enhanced in the *Low L* treatment (Figure 2B, Table 1), as well as nitrogen content of second generation leaves (Figure S2 in Supplementary Material). Only the *Low N* treatment had a strong negative effect on the plants in terms of reduced biomass production, lower effective quantum yield and increased C/N ratio (Table 1). However, the plants did not show changes in the photosynthetic rate as response to nutrient reduction (Figure 2A).

### Allocation of label derived ^13^C

**Figure 3:**
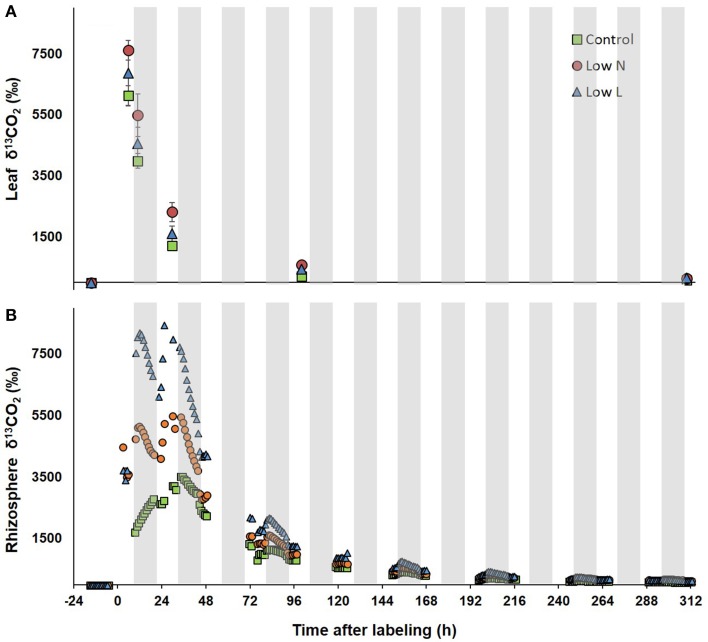
**δ^13^CO_2_ of leaves (A) and rhizosphere (B) during the chase period after pulse labeling**. δ^13^C of leaf respiration (*n* = 3 ± SE, after day 3 *n* = 2) was measured via in-tube incubation technique (Werner et al., 2007). Rhizosphere respired CO_2_ was measured in the soil compartment of one whole plant gas-exchange chamber per treatment. Gray areas indicate dark periods.

After 15 months we conducted a 4-h ^13^CO_2_ pulse labeling with a 13 days chase period. Five hour after labeling leaf respired CO_2_ was strongly enriched in ^13^C followed by a decrease of about 80% within 30 h with minor differences between treatments (Figure 3A). In contrast, differences between treatments were pronounced in ^13^C dynamics of rhizosphere respiration during the first 3 days after labeling (Figure 3B). *Low L* and the *Low N* plants rhizosphere respiration reached a first ^13^C peak in the first night and a second peak of similar size was measured 24 and 29 h after labeling, respectively. No distinct double peak was found in the control treatment, which showed the highest δ^13^CO_2_ values 33 h after labeling (Figure 3B). The half-life time of labeled C in leaf respiration substrate (estimated by δ^13^CO_2_ exponential decay functions) ranged between 11 and 14 h, whereas those from rhizosphere respiration ranged between 26 and 31 h (Table 2).

**Table 2:** **Carbon mean residence time (MRT), half-life time (t½) and coefficient of determination (R^2^) in respiratory substrate pool (calculated from exponential decay functions of δ^13^CO_2_)**.

	**Leaf respiration**			**Rhizosphere respiration**		
	**MRT (h)**	***t*½ (h)**	***R*^2^**	**MRT (h)**	***t*½ (h)**	***R*^2^**
Control	20.5	14.2	0.99	45.0	31.2	0.98
Low N	20.2	14.0	0.99	40.9	28.4	0.97
Low L	15.6	10.8	0.99	38.0	26.3	0.98

Parameters were calculated by fitting exponential decay functions (Equation 5) to leaf and rhizosphere respiration (Figure 3) beginning with the highest peak. All values were significant (P < 0.05).

**Figure 4:**
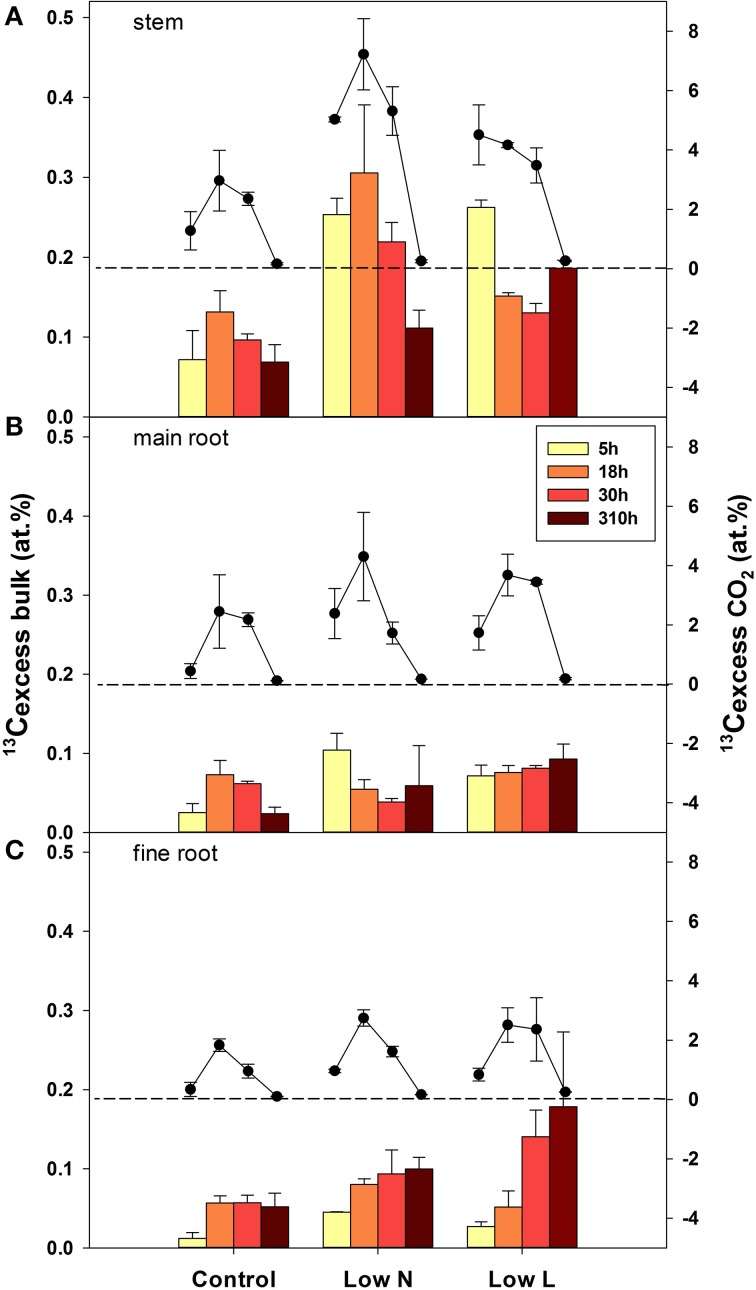
**Mean label derived ^13^C (^13^C_excess_), in bulk material (bars, left y-axis) and respired CO_2_ (circles, right y-axis) in stems (A), main roots (B), and fine root (C) over the four harvests during the chase period (*n* = 2± SE)**. The broken lines indicate zero on the right y-axis.

After pulse labeling plants were harvested four times during the chase period (5, 18, 30, 310 h =13 days). The isotopic compositions of bulk material form all tissue classes and respired ^13^CO_2_ from three tissues (stem, main root, and fine root) were analyzed. Overall, the highest label derived ^13^C enrichment (^13^C_excess_) in respiration of stem, main roots, and fine roots was found at the second harvest after 18 h (Figure 4). After 13 days the ^13^C label in respiration was very low, whereas the label derived ^13^C in organic matter, particularly in root material was still high or even reached a maximum. The velocity of C allocation to roots was slower in the *Low L* compared to the *Low N* treatment but resulted in a higher ^13^C_excess_ after 13 days. Both, the lowest ^13^C uptake and the lowest ^13^C loss (via respiration) were found in control plants (Figure 4).

**Figure 5:**
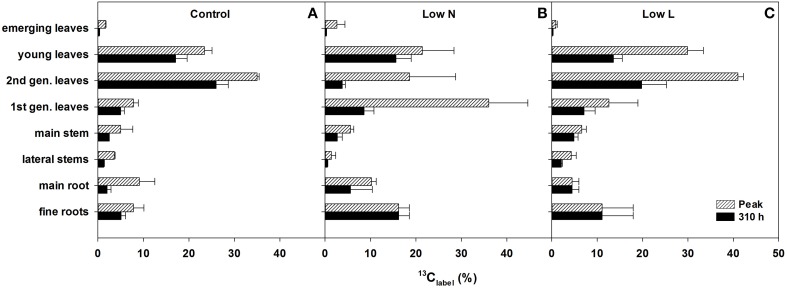
**Percentage of initially fixed ^13^C from label (^13^C_label_; Equation 4) in eight tissue classes**. Maximum measured ^13^C_label_ peak (hatched bars) and ^13^C_label_ present after 310 h (filled bars) of control **(A)**, *Low N* **(B)**, and *Low L* **(C)** treatment are shown (*n* = 2 ± SE). In case of equal length of the bars the highest value of the chase period was measured at the last harvest (310 h).

**Figure 6:**
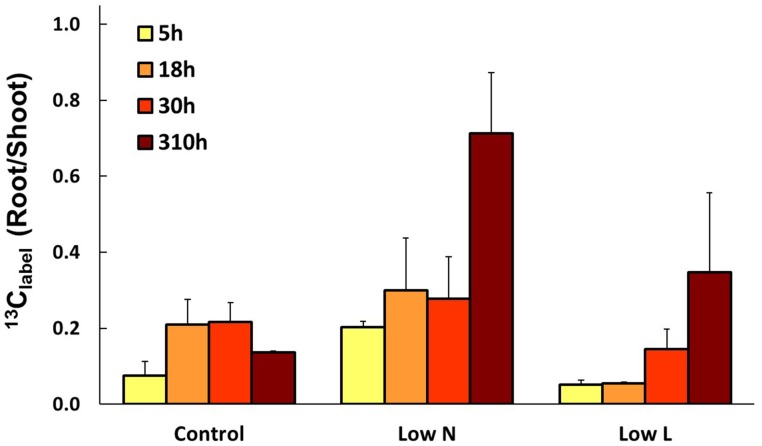
**Mean ratio of initially fixed ^13^C from label (^13^C_label_) in roots and shoots over the chase period (*n* = 2 ± SE)**.

We calculated the remaining fraction from initially fixed ^13^C from the ^13^C pulse (^13^C_label_; Equation 4) to compare the relative distribution within the plant and over time. Young leaves showed their highest ^13^C_label_-values rapidly in all treatments (after 5 h, data not shown), reaching 20–30% of initially fixed carbon from the whole plant (Figure 5). In control and *Low L* plants second generation leaves showed the highest proportion of ^13^C_label_. In contrast, first generation leaves were the major assimilating tissue class in the *Low N* treatment (Figure 5B). ^13^C_label_ in fine roots of *Low N* and *Low L* plants increased during the whole chase period up to 16 and 11%, respectively (Figure 5). Plants of both treatments predominantly invested in belowground tissues compared to the control (Figure 5). Compared to control, *Low L* plants invested 100% more C in the main stem at the expense of the younger leaf classes. The *Low N* treatment clearly showed reduced allocation to second generation leaves by c. 85% relative to control (Figure 5). In accordance with these results, the ratio of ^13^C_label_ of roots and ^13^C_label_ of shoots showed an increasing basipetal ^13^C translocation during the chase period in *Low N* and *Low L* plants (Figure 6). In the *Low N* treatment the ^13^C_label_ root/shoot ratio reached 0.2 within the first 5 h after labeling. This fast allocation was especially evident in main roots (Figure 4B).

**Figure 7:**
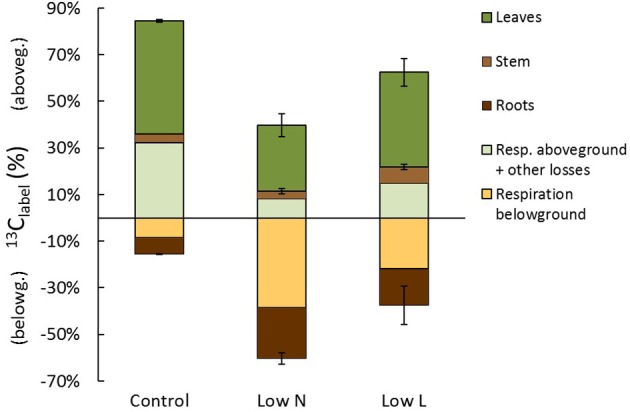
**Carbon allocation at the end of the chase period 13 days (310 h) after labeling**. The percentage of initially fixed ^13^C from label (^13^C_label_) separated into C allocation to aboveground (positive) and belowground (negative). Shown are C losses (respiration and other losses: *n* = 1) and C in plant tissues (in leaves, stems, and roots; *n* = 2 ± SE).

At the end of the chase period the ^13^C recovered in biomass was relatively equal in control, *Low N*, and *Low L* plants with 59, 53, and 63%, respectively (Figure 7). Control plants allocated 52% of ^13^C_label_ into aboveground tissues and only 7% into roots, whereas *Low N* plants incorporated 31% in above- and in 22% belowground (Figure 7). Carbon loss by rhizosphere respiration tended to be very high under low nitrogen conditions and low under control conditions. The total C translocation of recent assimilates to belowground (respiration and incorporation) was 15, 61, and 38% in control, *Low N*, and *Low L* plants, respectively. After subtracting biomass incorporation and rhizosphere respiration from initially fixed ^13^C the remaining fraction consists of aboveground respiration and other C losses. This fraction was remarkably large in control plants and small in *Low N* plants (Figure 7), which is consistent with the high root/shoot ratio (Figure 1D) and the low respiration rate on leaf level (data not shown) in the *Low N* treatment.

## Discussion

### Intraspecific variability in carbon allocation

In a meta-analysis, Poorter et al. (2012) concluded that plants have a lower ability to adjust allocation than to alter organ morphology, providing only little support for the importance of allocation in adaption of plants to their environment. In addition to morphological and physiological changes, we found pronounced differences in C allocation even in the root-shoot ratio, the simplest allocation parameter. This is remarkable as we focused on intraspecific variation of C allocation.

We found inverse patterns of C allocation within the main aboveground plant organs (leaves, stem). Compared to the control, plants of both treatments invested less C in younger leaf tissues but more in first generation leaves. Further, plants grown under nutrient limitation showed a slightly higher investment to main stems but a 60% lower allocation to lateral stems relative to the control. Only the allocation to main and fine roots was coherent within the treatments. Thus, our results demonstrate that there could be an evident loss of information when allocation within plants is summarized to changes in leaves, stems and roots. The investment in a specific leaf class (i.e., second generation leaves) could be shown to be very important for the development of spatial δ^13^C pattern in *H. halimifolium* (Wegener et al., 2010, 2015).

The higher SLA under light reduction is in accordance with results from previous studies. Poorter et al. (2012) found that shade increased SLA (on average 200%) much stronger than leaf mass fraction (LMF, 30%). We even found no significant changes in LMF in the *Low L* treatment compared to control but a significantly enhanced stem mass fraction (SMF) as a response to light reduction, which highly correlated with plant height over all plants (*R*^2^ = 0.69, *p* < 0.0001, data not shown). Overall, plants grown under light reduction showed enhanced SLA, leaf area and stem elongation but, remarkably, no significant increase in biomass allocation to aboveground. Indeed, *H. halimifolium* exhibits high phenological plasticity compared to other Mediterranean species (Zunzunegui et al., 2005; Werner et al., 2010). Previous experiments have shown a large plasticity in response to nutrient and shading treatments (Peperkorn et al., 2005). Within a species survey of very different plant functional types it revealed very interesting pattern in carbon metabolism (Priault et al., 2009) and carbon sources (Werner et al., 2009).

While thick and pubescence leaves are efficient to avoid photoinhibition and dehydration, particularly under field conditions in summer (Werner et al., 2002), these Mediterranean shrubs produce seasonal dimorphic leaves to adapt to changes in environmental conditions (Correia et al., 1992; Werner et al., 1999, 2001). The thinner leaves of Low L plants might have enhanced leaf internal light transmission and facilitate CO_2_ exchange, resulting in higher internal CO_2_ concentration (S1) thereby enhancing photosynthetic carbon gain/area.

Furthermore, compared to control plants, light reduction in *H. halimifolium* induced more C allocation to roots (c. 120%). This is possibly caused by the higher nutrient demand in those plants (Figure S2 in Supplementary Material).

The partitioning between C incorporation and C loss was relatively stable across treatments (37–47% C loss after 13 days), whereas the C fractions translocated to above- and belowground clearly differed between treatments. The strongly increased rhizosphere respiration in *Low N* could indirectly alter C metabolism by causing a higher rate of internal CO_2_ recycling (Bloemen et al., 2013). Aboveground respiration was calculated as the remaining proportion of initially fixed ^13^C from pulse labeling after subtracting incorporated and belowground respired ^13^C fractions. This fraction should mainly be respiration but also contains other C losses like fine root turnover, litter fall, abrasion of leaf waxes, root exudates, and emission of volatile organic compounds (VOC). Especially C loss through VOC production is likely as *H. halimifolium* was shown to be a strong VOC emitter (Jardine et al., 2014). Photorespiration is another important process which depends on illumination and internal CO_2_ concentration. However, the fraction of C loss caused by photorespiration could not be quantified in our study. Although it reduces C fixation, it is not traceable by ^13^C pulse labeling, which follows the fate of carbon that has been fixed successfully.

In our study light and nutrient reduction enhanced the coupling between photosynthesis and rhizosphere respiration, whereas drought was shown to reduce this coupling compared to control plants (Ruehr et al., 2009; Barthel et al., 2011). Plants of both resource limitations showed a double peak in δ^13^CO_2_ of rhizosphere respiration, similar to recent observations in pulse-labeling experiments in grasslands (Bahn et al., 2009) and beech saplings (Barthel et al., 2011). These findings were explained by transitory starch accumulation incorporating ^13^C during the labeling time window and subsequent re-mobilization at night, resulting in ^13^C re-enrichment of soil respiration with a certain time-lag. The absence of a clear double peak in control plant rhizosphere respiration is probably a result of the low C translocation rate to belowground tissues.

In addition to environmental conditions, the size and the developmental stage of a plant (ontogeny) could also influence allocation patterns (e.g., Reich, 2002). At the end of our experiment, plants grown under nutrient reduction had produced only one third of the biomass produced by control plants. However, our measurements during the treatment period show that allocation changes were not simply a function of plant size. The root-shoot ratio, LMF, SMF, and RMF of control plants differed from final *Low N*-values, even when control plants had the same biomass (c. after 6 months).

### Regulation of photosynthesis and C allocation

In case of “source regulation” of photosynthesis control plants should show the highest photosynthetic rate per leaf area as they had higher light and nutrient availability compared to the reduction treatments. In fact, although the nutrient reduction heavily reduced biomass production, the photosynthetic rate was hardly affected. Our results indicate that in *H. halimifolium* a decreased C demand (e.g., as a consequence of nutrient deficiency) is regulated by a reduction of C allocation to autotrophic tissues (i.e., less leaf area) rather than by a reduction of the photosynthetic rate. This is in contrast to results from other plant species where nitrogen deficient plants showed lower photosynthesis (e.g., Paul and Driscoll, 1997; Araya et al., 2010). Plants grown under light reduction even showed a 30–80% enhanced photosynthetic rate throughout the experiment when measured under the same light intensity as control plants (Figure 2). Further, they showed a considerably enhanced light-saturated photosynthesis compared to control. This indicates an up-regulation of the photosynthetic apparatus to avoid C starvation under low light conditions (e.g., Layne and Flore, 1995) and potentially a down-regulation of photosynthesis in control plants caused by endogenous factors. Possible explanations for the improved photosynthesis of plants grown under light reduction are: a higher internal CO_2_ concentration (due to changes in leaf morphology), higher photosynthetic efficiency (reflected in higher ΔF/Fm′) and a more efficient light use in the canopies of Low L plants. Through these adaptions combined with higher leaf area, plants grown under a 57% light reduction reached the same final biomass as control plants. In the second half of the experiment control plants were probably sink limited compared to the other treatments and decreased photosynthetic rates. This is in accordance with the results from our labeling experiment, where the control plants fixed the lowest amount of ^13^CO_2_. In control plants the fraction of incorporated C at the end of the chase period was clearly lower than the maximum value in each tissue class (Figure 5), which further indicates a lack of strong C sinks. In contrast, plants grown under light and nutrient reduction showed strong allocation to sink tissues (roots and stems) and therefore a higher C demand compared to control plants. Together, these results support the concept that plants show a high plasticity in active adjustment of morphology, physiology and C allocation to optimize carbon gain under different environmental conditions.

Although the amount of label derived ^13^C declined strongly in the available C pools (i.e., decline of ^13^CO_2_ in respiration) in the first days, some heterotrophic tissues showed steady or even increasing values of label derived ^13^C in bulk material until 13 days after labeling in both treatments. This pattern was most pronounced in the tissues with enhanced C gain compared to control (roots in both and stems in the *Low L* treatment). Several studies found half-life times ranging from several months to years in fine roots (Joslin et al., 2006; Endrulat et al., 2010; Keel et al., 2012). Together, these results indicate a very low rate of C loss (respiration and other losses) once C is allocated to storage or structural pools in these growing heterotrophic tissues. Our results further indicate that in those tissues respiration must be dominantly fuelled by recent assimilates. This is reflected in the shorter MRT in the respiratory substrate pool and the higher proportion of recovered ^13^C (^13^C_label_) in rhizosphere respiration in case of nutrient and light limitation. We propose that this reduction of the C reflux from storage and structural C pools could be a control mechanism for C acquisition by heterotrophic tissues (see Farrar and Jones, 2000). In case of steady respiration rates this mechanism would lead to a fast increase in C sink strength of the growing tissue even before biosynthesis itself causes a higher C demand. Nogués et al. (2014) found an analogous interspecific relationship where fast-growing plants used five times more recently assimilated C for respiration than slow-growing plants.

## Conclusions

The adjustment in allocation pattern was partly inconsistent within the main plant organs (old vs. young leaves, main stem vs. lateral stems). Thus, our results show that the common separation of plant tissues in only three classes, i.e., root, shoot and leaf, can disguise allocation changes. Further, our results indicate a high intraspecific plasticity in both, C allocation as well as in morphological and physiological traits under varying environmental conditions. The pronounced treatment effects were not reflected in the photosynthetic rate. Therefore, our study supports the idea that plants, within certain limits, actively adjust their performance (by morphological and physiological adaptions) to reach an optimal rate of C fixation. Finally, the results indicate that growing heterotrophic tissues strongly reduce the C reflux from storage and structural C pools and therefore enhances the fraction of recent assimilates used for respiration. This shift could alter the C balance between sources and sink tissues and is potentially a regulatory mechanism for C uptake and translocation.

### Conflict of interest statement

The authors declare that the research was conducted in the absence of any commercial or financial relationships that could be construed as a potential conflict of interest.
