# Supplementary material for: High intraspecific ability to adjust both carbon uptake and allocation under light and nutrient reduction in Halimium halimifolium L
Source: Front Plant Sci. 2015 Aug 7;6:609. doi: 10.3389/fpls.2015.00609 (PMC4528176; doi:10.3389/fpls.2015.00609)

# Supplementary material

**S1** Gas exchange parameters measured at growth conditions (370µmol m^-2^ s^-1^ for control and Low N and 160µmol m^-2^ s^-1^ for Low L plants) during the experiment (sampling points at 4, 6 and 12 months). Low L plants were further measured at 370 µmol m^-2^ s^-1^ (below the broken line). Data are means (± SE; n=4-5). A - assimilation rate; c_i_/c_a_ - intercellular to ambient CO_2_ concentration; gH_2_O - stomatal conductance for water vapor;

| Harvest/ Months | Treatment | Light  µmol m^-2^ s^-1^ | A_leaf_  µmol m^-2^ s^-1^ | *±SE* | c_i_/c_a_ | *±SE* | gH_2_O  mmol m^-2^ s^-1^ | *±SE* |
| --- | --- | --- | --- | --- | --- | --- | --- | --- |
| 4 | Control | 370 | 5.8 | *0.3* | 0.49 | *0.02* | 48.2 | *4.4* |
| 6 | Control | 370 | 3.9 | *0.5* | 0.41 | *0.03* | 26.8 | *3.0* |
| 12 | Control | 370 | 2.7 | *0.5* | 0.47 | *0.03* | 21.7 | *2.8* |
| 4 | Low N | 370 | 5.3 | *0.4* | 0.66 | *0.02* | 67.1 | *7.3* |
| 6 | Low N | 370 | 3.8 | *0.7* | 0.47 | *0.05* | 27.0 | *7.1* |
| 12 | Low N | 370 | 3.2 | *0.7* | 0.63 | *0.04* | 36.3 | *11.3* |
| 4 | Low L | 160 | 3.6 | *0.2* | 0.63 | *0.04* | 42.4 | *4.5* |
| 6 | Low L | 160 | 3.1 | *0.1* | 0.59 | *0.03* | 29.8 | *3.9* |
| 12 | Low L | 160 | 3.0 | *0.4* | 0.58 | *0.05* | 35.3 | *8.0* |
| 4 | Low L | 370 | 7.6 | *0.4* | 0.58 | *0.03* | 80.3 | *16.6* |
| 6 | Low L | 370 | 5.2 | *0.6* | 0.43 | *0.04* | 38.6 | *7.4* |
| 12 | Low L | 370 | 4.9 | *0.7* | 0.54 | *0.02* | 37.3 | *9.9* |

**S2** Nitrogen content (%) of second generation leaves (see material and methods for definition) during the 15 months of treatment (n = 3-8 ± SE).


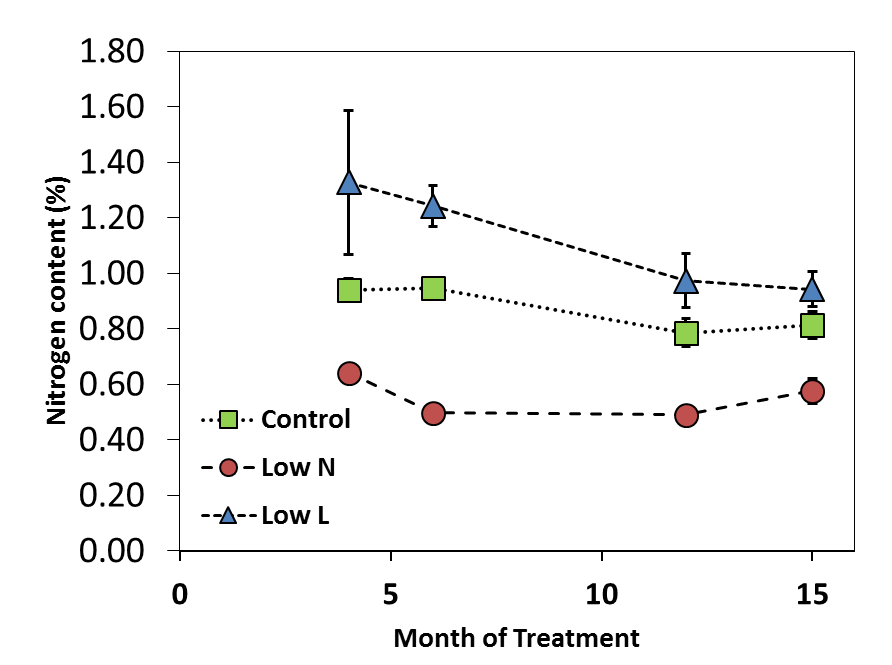

Supplement: Supplementary file 1 [file Table1.DOCX]
